# Which Models Are Appropriate for Six Subtropical Forests: Species-Area and Species-Abundance Models

**DOI:** 10.1371/journal.pone.0095890

**Published:** 2014-04-22

**Authors:** Shi Guang Wei, Lin Li, Zhen Cheng Chen, Ju Yu Lian, Guo Jun Lin, Zhong Liang Huang, Zuo Yun Yin

**Affiliations:** 1 Guilin University of Electronic Technology, GuiLin 541004, China; 2 South China Botanical Garden, the Chinese Academy of Sciences, GuangZhou, 510650, China; 3 Changjiang Water Resources Protection Institute, WuHan, 430051, China; 4 Guangdong Forest Research Institute, GuangZhou, 510520, China; Institute of Botany, Chinese Academy of Sciences, China

## Abstract

The species-area relationship is one of the most important topic in the study of species diversity, conservation biology and landscape ecology. The species-area relationship curves describe the increase of species number with increasing area, and have been modeled by various equations. In this paper, we used detailed data from six 1-ha subtropical forest communities to fit three species-area relationship models. The coefficient of determination and *F* ratio of ANOVA showed all the three models fitted well to the species-area relationship data in the subtropical communities, with the logarithm model performing better than the other two models. We also used the three species-abundance distributions, namely the lognormal, logcauchy and logseries model, to fit them to the species-abundance data of six communities. In this case, the logcauchy model had the better fit based on the coefficient of determination. Our research reveals that the rare species always exist in the six communities, corroborating the neutral theory of Hubbell. Furthermore, we explained why all species-abundance figures appeared to be left-side truncated. This was due to subtropical forests have high diversity, and their large species number includes many rare species.

## Introduction

The species-area relationship (SAR) is among the well known and most studied patterns in ecology [1], [2], [3], [4], [5], [6], and it is important to both our basic understanding of biodiversity and ability to conserve biodiversity [7]. The SAR describes the increasing number of species (*S*) as area (*A*) increases; when plotted, the SAR is, in theory, a monotonically increasing curve whose slope is steep at first but gradually becomes nearly flat. In reality, SAR curves have a variety of shapes depending on different sampling strategies, biases, and community parameters. The SAR has been used to determine the minimal sampling size of a particular community [8], [9], to estimate species richness [10], [11] or species extinction as a result of habitat loss and fragmentation [12], [13], [14] and to calculate the effective size of natural reserves for long-term conservation of biodiversity [15], [16].

The SAR has been explained by two ecological hypotheses: (1) the habitat heterogeneity hypothesis predicts that, as area increases, habitat heterogeneity increases, and the total number of species also increases; (2) the area *per se* or equilibrium hypothesis invokes colonization–extinction dynamics which postulates that, as area increases, relative rates of colonization increases while rates of extinction decreases, and therefore the total number of species increase with area [4]. However, habitat heterogeneity and area are often closely interdependent, and therefore their relative influences are difficult to disentangle [17], [18]. Equal sample sizes, such as sampling equally sized areas, should reduce effects of area, which, in turn, may be correlated with habitat heterogeneity [19], [18], [20].

Traditionally, the functional forms of the SAR have been modeled with convex-upward functions lacking an asymptote, such as the power [21], exponential [22], [23], [24], logarithmic [22], [23], and logistic [25], [26], [27] functions, as well as the random-placement model [28]. More convex and asymptotic models as well as sigmoid models have also been used [29]. Overall, it seems that the power model represents a reasonably good approximation of the SAR, and for intermediate spatial scales [21], [4], [30].

Conventional reasoning behind the SAR is based on another fundamental ecological pattern, the species–abundance distribution (SAD), which describes how individuals are distributed among species [31]. He and Legendre [27]pointed out that area, species-abundance (SA), spatial distribution, and species richness have been central components of community ecology, and mathematically explore the interrelationships among these components. Among these, the SA pattern is so fundamental that Sugihara referred to it as “minimal community structure” [32].

There are two main types of models that have been suggested to characterize the SA pattern in biological communities [33], [34], [35]. One type is the rank-abundance curve [36], [37], or dominance-diversity curve [38], [39], which is described mainly by the broken stick model or the random niche-boundary hypothesis of MacArthur and Wilson [15] as well as the geometric series [40]. The second type is the SA distribution, which has been commonly modeled by the logseries distribution [24] or the lognormal distribution [41]. Fisher *et al.* [24]found the logseries distribution by assuming that the parameter *k* of the negative binomial distribution is close to zero; it was subsequently found that a great deal of data also fitted the logseries model [42], [43], [3]. The lognormal distribution has been derived through multiple routes, most recently from neutral community theory assuming identical species [44], [45]. The lognormal model is a more general model of SA patterns in natural communities [46] and can therefore be fitted to all data that also fit the logseries model well. Hubbell’s [45] theory suggested that Fisher’s logseries describes the SAD of a metacomunity, while Preston’s lognormal model describes the SAD of a local community. Yin *et al.* [35] tested two alternative models, the so-called logcauchy and log-sech models which have simpler functional forms than the commonly used lognormal model [47]; these two alternative models fitted the observed SADs better than the lognormal model.

Tropical forests are generally considered to be ecological systems well suited for species-area and spatial-aggregation research [48], [28]. Subtropical forests display a relatively high diversity of tree species, therefore providing suitable conditions for ecological analyses. Moreover, it is urgent to study their ecology, because the area of subtropical forests has recently been rapidly reduced while the survived forests have been degraded and fragmented [49]. Studying trees has the obvious advantage, because their sedentary life history makes them relatively easy to relocate, and most them can also be identified because their taxonomy is rather well-resolved.

Based on a detailed data set from six subtropical forests, in this study we intend (1) to examine the validity of the random-placement, power law and logarithm model for subtropical forests, to fit the corresponding SAR relationships and then compare their fitted results to find the appropriate model to express the SAR of subtropical forests; (2) to focus on the second type of SAD models, i.e., to fit the lognormal, logseries and logcauchy distributions, and to find the suitable SAD models for subtropical forests; and (3) to compare species richness among different communities, and based on SAR models to explain ecological changing processes of the subtropical forests based on fitted SAR models. We also discuss the influence of different sampling sizes and sampling methods on model fitting. This study thus contributes to our understanding of SAR and SAD in subtropical forests.

## Methods

### Ethics Statement

No specific permits were required for the described field studies. Our study site (six 1-ha plots in the subtropical forests of southern China) is owned by the Chinese government and managed by South China Botanical Garden, Chinese Academy of Sciences. We can do our research works freely in these plots under the Regulations of the People’s Republic of China on Nature Reserves. Our field studies did not involve endangered or protected species.

### Sampling Tree Communities

For this study, we censused trees in six 1-ha plots in the subtropical forests of southern China (Table 1). In each community, each woody stem ≥1 cm diameter at breast height (DBH) was identified to species. Community A was sampled within the Maoershan National Nature Reserve (110°30′E, 25°56′N), which belongs to the monsoon evergreen broad-leaved forest. Community B was sampled within Nanling Nature Reserve (112°56′–113°4′E, 24°30′–24°48′N), which belongs to broad-leaved forest. Communities C and D were sampled within the Dinghushan Biosphere Reserve (112°30′39″–112°33′41″E,23°09′21″–23°11′30″ N), which contains monsoon evergreen broad-leaved, needle and broad-leaved mixed forests. Communities E and F were sampled within the Jinggangshan Nature Reserve (114°05′–114°23′E,26°22′–26°48′ N), which contains broad-leaved, needle and broad-leaved mixed forests.

**Table 1:** A list of the six 1-ha communities of subtropical forest (see Methods for details), listing tree density measured as stem density and tree diversity measured as observed and estimated species richness for each community.

Community	Plot name	Forest type	Stemdensity	Observed speciesrichness	Estimated speciesrichness
A	Maoershan	Monsoon evergreen broad-leaved forest	2894	89	93.6
B	Nanling	Broad-leaved forest	3326	165	229.7
C	Dinghushan plot 1	Monsoon evergreen broad-leaved forest	3609	95	139.7
D	Dinghushan plot 2	Needle and broad-leaved mixed forest	4003	63	99.1
E	Jinggangshan plot 1	Broad-leaved forest	3950	117	160.7
F	Jinggangshan plot 2	Needle and broad-leaved mixed forest	2440	95	121.8

Estimated species richness was calculated by randomized sampling order of each sample using the software *EstimateS* (see Methods for details). We assume that the estimated species richness is close to the total, or true, species richness [29].

### SAR Models

The empirical study of SAR dates back to H. C. Watson, who presented the first known species-area curve for Great Britain’s vascular plants in 1859 [50]. Watson found a linear relationship between the logarithm of the number of species present and the logarithm of the area sampled, over areas ranging from a square mile to all of Great Britain. This is the most common pattern found on regional scales within relative homogeneous landscapes [22], [4]. The relationship is given by the so-called *power* function:
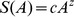
(1)where *S* is the total number of species encountered in the geographical area *A*, *c* is the parameter characterizing the specific biogeographical region and *z* is the parameter which is specific to the sampled community.

The second SAR model is the *logarithm* model, which is transformed from the model mentioned by Buys *et al.* [51]. Liu *et al.* [52] used the data of five plots located in the temperate zone to fit ten species-area models and showed that the following logarithm model provided excellent fits:
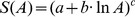
(2)where *S* (*A*) is the total number of species encountered in the geographical area *A*, *a* is the species number in the area unit, *b* is a parameter independent from area, which is considered a measure of spatial heterogeneity and *c* is a fitted constant.

The third model is Coleman’s “zeroth-order”, *random-placement* theory of species-area curves. The random-placement model is suite for natural random distribution that is underlying processes. It describes the slow-rapid-slow accumulation of new species in a region. In other words, it is of the logistic type [53]. Consider a region of total area *A*_0_ within which individuals of various species are located. Assume that there are *N* species in the area *A*_0_, and that the *i*-th species is represented by *n_i_* individuals. Consider any sub-region of area *A*<*A_0_*. Under the assumption of independent, random placement of individuals, the probability that a given member of the *i*-th species does not reside in a sub-region of size *A* is simply (1-*A*/*A_0_*). Similarly, the probability that all members of species *i* lie outside of *A* is given by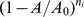. Thus, the probability that at least one member of the *i*-th species resides in the sub-region *A* is 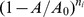 which, in turn, yields an expression for the mean number of species in *A*, denoted *S*(*A*) [53]:
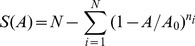
(3)


### SAD Models

Preston [41] adopted the *lognormal* model to describe a biological community with few dominant and rare species, but with numerous medium-abundant species. His model is:
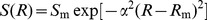
(4)where *S*(*R*) is the species number of the *R*th octaves; *R*_m_ the modal (peak) octave; *S*_m_ the number of species in the *R*_m_th octave (or “height” of the distribution curve) while *a* is a constant describing the amount of spread of the distribution [41], [54]. In order to fit the field data to the lognormal distribution, we adopted the linear regression model 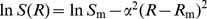 to calculate the parameters.

The second SAD model is the *Logcauchy* distribution model [35]:
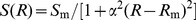
(5)


The means of parameters are the same as in the lognormal model.

The third SAD model is the *logseries* model. Fisher *et al.* [24] first used the logseries model to fit the SAD of Malayan butterflies and Lepidoptera. It is one of the two well known SAD models (the other one being the lognormal model). The number of species with *n* individuals in a community is:
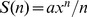
(6)where *a* and *x* are two parameters, *a*>0 and 0<*x*<1 (in most cases *x*>0.9). However, these two parameters are not independent. The parameter *a* is called Fisher’s *α* diversity index. It was widely used in 1960’s and 1970’s as a diversity index and has found renewed interest through the recently developed neutral theory of biodiversity [45]. In Hubbell’s theory, *a* is a fundamental biodiversity parameter. He and Hu [55] further showed that Hubbell’s fundamental biodiversity parameter is a function of Simpson’s diversity.

### Data Analysis

When we calculated the SADs, we adopted the octave method to divide groups of *R* values. We let each octave’s midpoint to be equal to twice that of the preceding octave’s midpoint. That is *R* = 2*^x^* (*x* = 0, 1, 2, …),e.g., 1, 2, 4, 8, …. In this way, the midpoint of each group is 1.5, 3, 6, …. If the individual number of a species is found on the edge of both group’s octaves, that is to say it has 2*^x^* individual, then we included it in the(2*^x^* ^-1^, 2*^x^*)octave, otherwise in the (2*^x^*, 2*^x^* ^+1^) octave. This method is equal to calculating the logarithm of the individual number of each species using the base 2.

The total (or true) species richness of each community was calculated using the software *EstimateS* 8.2 [56]. Based on the research done by Walther and Moore [29], Wei *et al.* [57] found that, for areas below 7 ha, the least biased estimator is the second-order jackknife estimator (*Jack2*). Using the default settings of *EstimateS* to calculate Jack2, which are 50 randomized runs to estimate species richness, i.e. using 50 different, randomly chosen sampling orders to calculate the estimate of total species richness.

Curve fitting and significance tests were performed in the R2.3.1 platform downloaded from the R Foundation for Statistical Computing, Vienna, Austria (ISBN 3-900051-07-0, http://www.R-project.org). The Levenberg-Marquardt least squares nonlinear regression was used to fit the power, logarithm, lognormal, logseries and logcauchy models. The *F* ratio of ANOVA was used as the best-fit criterion of the SAR models. The better model is the one with the lowest *P*-value (i.e., probability under the null hypothesis) [26].

we also computed the adjusted coefficient of determination (*R_a_^2^*) for the goodness-of-fit of SAR and SAD models, the larger *R_a_* ^2^ was, the better the fitted model was [58], [59]. This coefficient was calculated as:
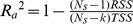
where *RSS* is the residual sum of squares, *TSS* is the total sum of squares, *N_S_* is the number of sampling intensities, and *k* is the number of parameters in a model. This coefficient is more suitable than the usual coefficient of determination *R^2^* in that it takes into account the respective numbers of degrees of freedom of the numerator and denominator [60].

## Results

### Tree Density and Diversity

Measures of tree density and diversity per hectare varied widely between the six communities (Table 1), ranging from 2440 to 4003 stems (mean ± S.E. = 3370.3±250.8), 63 to 165 observed species (mean ± S.E. = 104.0±14.1), and 93.6 to 229.7 estimated species (mean ± S.E. = 140.8±20.5).

Observed and estimated species richness resulted in broadly similar rankings of the six communities which is reflected in these two diversity measures being significantly correlated (Spearman rank correlation, *n = *6, *Z*-value = 2.08, *P*-value = 0.04). However, there were interesting exceptions: first, communities C and F were tied in observed species richness, but community C had a much higher estimated species richness than community F and, second, community A had a higher observed species richness but lower estimated species richness than community D. These inconsistencies and the fact that the estimated values are significantly higher than the observed values (One-sample sign test, *n* = 6, *P*-value = 0.03) further support the view that observed species richness is a negatively biased measure of total species richness (see Discussion). Therefore, we do not consider observed species richness from hereupon.

Ranking of communities by estimated species richness was not associated with forest type, with the ranking from lowest to highest estimated species richness being: monsoon evergreen broad-leaved forest, needle and broad-leaved mixed forest, needle and broad-leaved mixed forest, monsoon evergreen broad-leaved forest, broad-leaved forest and broad-leaved forest. Given this particular sequence and the same sample size, no trend can possibly be discerned between forest types.

Likewise, ranking of communities by number of stems per ha was not associated with forest type, with the ranking from lowest to highest tree density being: needle and broad-leaved mixed forest, monsoon evergreen broad-leaved forest, monsoon evergreen broad-leaved forest, monsoon evergreen broad-leaved forest, broad-leaved forest and needle and broad-leaved mixed forest. Given this particular sequence and the same sample size, no trend can possibly be discerned between forest types.

Finally, there was no correlation between number of stems and estimated species richness (Spearman rank correlation, *n* = 6, *Z*-value = 0.32, *P*-value = 0.75).

### Species-area Relationships

**Figure 1:**
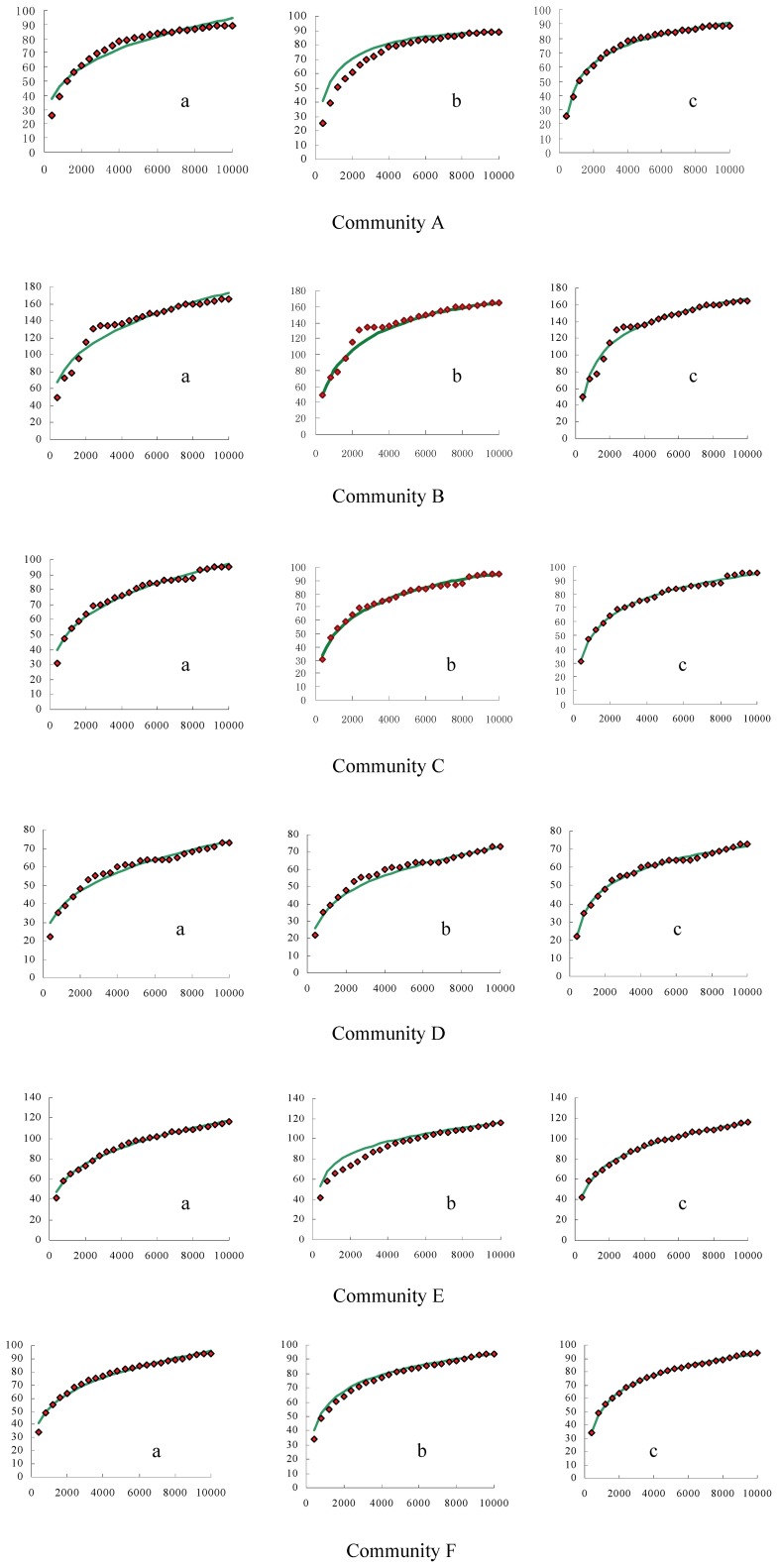
Species-area relationships in six subtropical forest communities (see Table 1), shown in successive rows (observed number of species: red diamonds; fitted model curves: green line). The left column (a) is simulated using the power model, the middle column (b) using the random-placement model, and the right column (c) using the logarithm model (for parameter values, see Tables 2 and 3). The *x*-axis represents the area (m^2^), and the *y*-axis represents the number of species.

Using the *F*-test, each of the three SAR models fitted highly significantly with the observed numbers by randomized sampling as well as sequential sampling (Tables 2 and 3, respectively). This good fit can also be observed graphically for each of the plotted species-area relationships (Fig. 1).

**Table 2:** The fitted results of SAR models for each of the six communities (see Table 1) using sequential sampling order of each sample (see Methods for details).

Model	Community	Simulative equations	F-value	*P*-value	*R_a_^2^*
Power	A	*S = *6.862*×A* ^0.285^	337.4609	<0.001	0.9362
	B	*S = *11.614*×A* ^0.293^	315.9296	<0.001	0.9321
	C	*S = *7.518*×A* ^0.278^	12535.55	<0.001	0.9774
	D	*S = *5.549*×A* ^0.281^	605.3688	<0.001	0.9634
	E	*S = *8.825*×A* ^0.281^	2789.154	<0.001	0.9918
	F	*S = *8.731*×A* ^0.260^	1276.937	<0.001	0.9823
Logarithm	A	*S* = (−1199.062+218.530×ln(*A*)) ^0.673^	1296.167	<0.001	0.9844
	B	*S* = (−1016.356+193.021×ln(*A*)) ^0.772^	404.593	<0.001	0.9735
	C	*S* = (−112.961+25.192×ln(*A*)) ^0.952^	1883.335	<0.001	0.9942
	D	*S* = (−51.339+12.067×ln(*A*))^1.046^	1292.309	<0.001	0.9787
	E	*S* = (−4.155+2.188×ln(*A*)) ^1.713^	3590.790	<0.001	0.9969
	F	*S* = (−117.760+27.171×ln(*A*)) ^0.93^	9144.175	<0.001	0.9988

**Table 3:** The fitted results of SAR models for each of the six communities (see Table 1) using randomized sampling order of each sample (see Methods for details).

Model	Community	Simulative equations	F-value	*P*-value	*R_a_^2^*
Power	A	*S = *6.228*×A* ^0.294^	561.376	<0.001	0.9606
	B	*S = *3.166*×A* ^0.433^	2481.539	<0.001	0.9908
	C	*S = *3.678*×A* ^0.355^	2890.841	<0.001	0.9921
	D	*S = *2.377*×A* ^0.357^	5835.481	<0.001	0.9961
	E	*S = *9.097*×A* ^0.278^	1328.496	<0.001	0.9830
	F	*S = *8.777*×A* ^0.259^	1274.382	<0.001	0.9823
Logarithm	A	*S* = (−374.208+72.061×ln(*A*)) ^0.795^	3264.962	<0.001	0.9966
	B	*S* = (−9.287+2.539×ln(*A*)) ^1.934^	14226.910	<0.001	0.9992
	C	*S* = (−7.909+2.431×ln(*A*))^1.703^	56109.664	<0.001	0.9998
	D	*S* = (−0.778+0.665×ln(*A*))^2.470^	33600.000	<0.001	0.9997
	E	*S* = (−118.654+26.893×ln(*A*)) ^0.977^	31513.588	<0.001	0.9997
	F	*S* = (−127.169+29.088×ln(*A*)) ^0.918^	81634.628	<0.001	0.9999
Random-placement	A		46.165	<0.001	0.8543
	B		141.806	<0.001	0.9623
	C		1109.576	<0.001	0.9899
	D		10.669	<0.001	0.9678
	E		81.376	<0.001	0.9006
	F		349.647	<0.001	0.9739

Using the adjusted coefficient of determination (*R_a_^2^*-value) for comparison (Tables 2 and 3), the logarithm model had the better fit among the three models (mean = 0.9878 for sequential sampling, mean = 0.9992 for randomized sampling), with three out of six of its *R_a_^2^*-values above 0.99 for randomized sampling. The power model was the second better model (mean = 0.9639 for sequential sampling, mean = 0.9842 for randomized sampling) while the random-placement model performed worst (no sequential sampling performed, mean = 0.9415 for randomized sampling). Because the random-placement model performed worst, if we average over all results, randomized sampling (*n* = 18, mean = 0.9749) performed worse than sequential sampling (n = 12, mean = 0.9758). However, once we exclude the random-placement model because it was not used for sequential sampling, we can compare the results from only the logarithm and power models; we then observe that randomized sampling (*n = *12, mean = 0.9917) performed better than sequential sampling (*n* = 12, mean = 0.9758). We argue that this better performance is due to randomized sampling smoothing the resulting SA curves (see Discussion).

The SARs (Fig. 1) illustrate that communities D, E and F were fitted very well beyond area sizes of about 4000–5000 m^2^. Communities C and F fitted well using the random-placement model at all area sizes while the communities C, E and F were fitted well with the logarithm model. Community A fitted worse than the other communities for all three models. All communities fitted well to the random-placement model beyond area sizes of about 7000 m^2^, suggesting that the random-placement model describes SARs better at larger scales.

### Species-abundance Distributions

**Figure 2:**
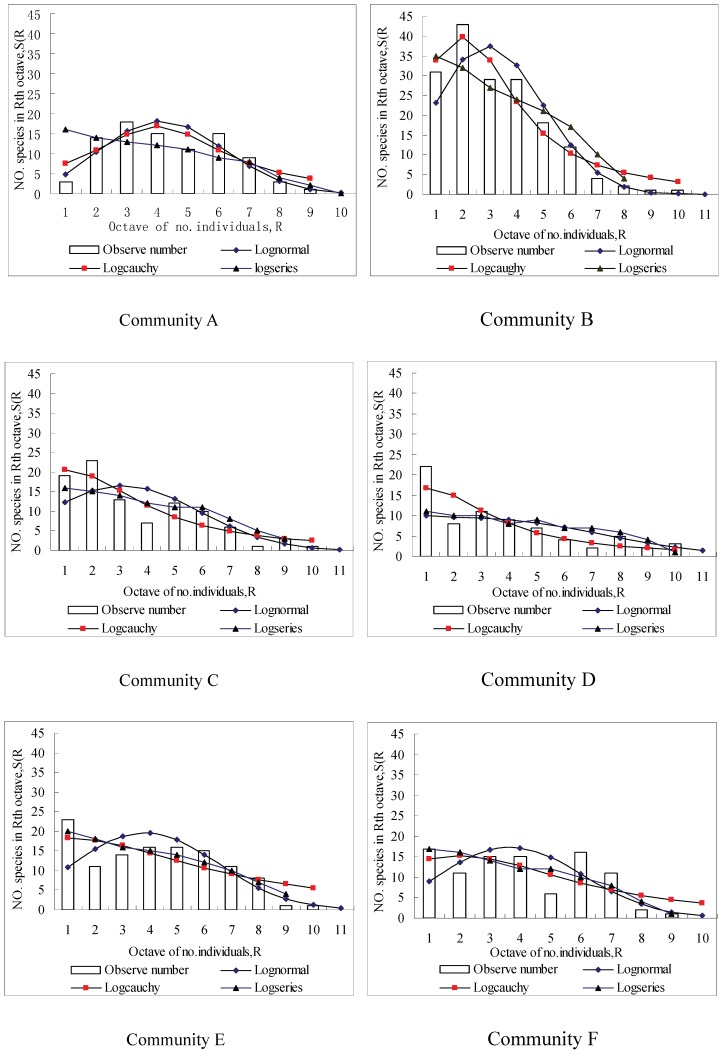
The species-abundance relationships simulated by the lognormal, logcauchy and logseries model (for parameter values, see Tables 4 and 5) for each of the six communities (see Table 1).

All the SA data sets were statistically fitted using the three SAD models (Tables 4–5) with each fit also displayed graphically in Fig. 2. Using the coefficient of determination (*R_a_^2^*) for comparison, the mean *R_a_^2^*-values over the six communities were 0.738, 0.648 and 0.586 for the logcauchy, lognormal and logseries model, respectively. This ranking is also evident in that the logcauchy had two *R_a_^2^*-values above 0.8 and two above 0.7, while the lognormal only had two above 0.8 and the logseries one above 0.8 and one above 0.7. Therefore, using *R_a_^2^*-values as criteria, the logcauchy model performed better than other two models.

**Table 4:** The fitted parameters and results for the lognormal and logcauchy models (see Methods for details).

Model	Community	Parameter			*R_a_^2^*
		*S*_m_	*a*	*R*_m_	
Lognormal	A	18.220	1.103	4	0.805
	B	37.521	0.756	3	0.895
	C	16.516	0.869	3	0.653
	D	10.121	0.879	1	0.453
	E	19.527	1.045	4	0.602
	F	17.067	0.988	4	0.481
Logcauchy	A	16.789	0.373	4	0.709
	B	39.7640	0.420	2	0.942
	C	20.693	0.230	1	0.838
	D	16.681	0.344	1	0.727
	E	18.224	0.168	1	0.620
	F	15.204	0.219	2	0.591

**Table 5:** The fitted parameters and results of the *χ* ^2^ tests for the logseries model (see Methods for details).

Model	Community	Parameter		*R_a_^2^*
		*x*	*a*	
Logseries	A	0.994	17.379	0.223
	B	0.989	37.889	0.833
	C	0.995	17.883	0.697
	D	0.997	12.335	0.463
	E	0.996	21.477	0.704
	F	0.993	18.811	0.595

The mean *R_a_^2^*-values of the six communities A–F were 0.579, 0.890, 0.729, 0.548, 0.642 and 0.556, respectively; thus, the best average fit for three SAD models was for community B and the worst for community D.

The three SAD models were graphically fitted using the SA data sets (Fig. 2). The results showed that the left side of each of the three distributions was apparently truncated for each of the six communities, which suggests that many rare species exist within these communities which were not detected within our samples.

## Discussion

### Tree Density and Diversity

The six sub-tropical forest communities displayed a wide range for stem density and estimated species richness (by a factor of 1.64 and 2.45, respectively). For comparison, species richness in three 1- ha plots of tropical seasonal rainforest in south-west China varied only by a factor of 1.26 and 1.61 for trees and treelets, respectively [61], [62]. This large variation among the six plots cannot be explained by forest type as the largest variation for stem density is within the needle and broad-leaved mixed forest type (Table 1). Currently, we cannot ascribe any factors responsible for such large variation, but latitude, altitude, topography and development history of each community is different, therefore, a combination of habitat heterogeneity and/or evolutionary- historical factors may be responsible for these differences [63], [64].

Surprisingly, neither stem density nor estimated species richness seemed to be influenced in any way by forest type. However, given the insufficient sample sizes for each forest type, not much should be read into this result. We are surprised that no correlation was found between stem density and estimated species richness, as there is often such a positive relationship between the number of individuals and the number of species [65].

Our results further support the view that observed species richness is a negatively biased measure of total species richness. This is the logical consequence of the shape of the SA curve if area is substituted for sampling effort. As sampling effort increases, species richness will increase, so it logically follows that insufficient sampling effort will lead to a negatively biased estimate of total species richness (also called observed species richness). Therefore, species richness estimators (as, for example, implemented in the program *EstimateS* or *SPADE*) need to be used to correct for this inherent sampling bias [66], [29]. The resulting estimated species richness is generally much closer to the total species richness than the observed species richness.

### Species-area Relationships

The question of the relationship between area and number of species is one of the oldest questions ecologists have posed [67], and the SAR is one of the most important relationships when investigating this relationship as well as related problems involving species diversity, conservation biology and landscape ecology [68], [69], [70], [4], [71]. Many models have been put forward to describe the SAR [29], three of which we applied in this study.

Every one of the tested three SAR models (power, logarithm, random-placement) fitted very well to the subtropical community data, with minor differences (see Results). Comparing the fits of these three models using mean *R_a_^2^*-values showed slightly better average fits for the logarithm model than for the power and the random-placement model, a result supported by Wei *et al.* [57].

Plotting the SAR for each community and visually comparing the fitted models gives us some interesting insights into the effect of sampling size (in our case, size of sampled area) on the goodness of fit of each model (Fig. 1). While there are examples where the respective model fits the real data very well for all sampling sizes (e.g., community F fitted by the logarithm model), which is reflected by very high corresponding *R_a_^2^*-values, other examples reveal that the fit is only good for some sampling sizes, with *R_a_^2^*-values also varying among different communities. In most cases, the fit is better for intermediate to high sampling sizes and worse for small sampling sizes. Random sampling effects usually have a greater proportional effect at small sampling sizes, which may explain why the real data curves do not conform so well to the model curves at lower sampling sizes. Randomization of sampling order usually removes these sampling effects, and therefore the resulting smoothed curves usually display better model fits even at low sampling sizes.

Several authors have argued that the performance of SAR models is dependent on the sampling size [26], [72]. For example, He &Legendre [27] suggested the power model best fits for small to intermediate sampling sizes, the exponential model best fits for small samples, and the logistic model is the best for small to large scales (250,000 m^2^). And consequently, that there is no model that is universally best, but that each model’s performance depends on sampling scales and SADs [26]. Because the underlying SADs are different for different communities, Tjørve [73] claims that one should not expect the same SAR model to be the best fit for both sample area (census patches) data sets and isolate (habitat patches or islands) data sets [73], or across different scales (see also [74]).

The SAR curves in this study showed similarity to those in Condit’s [75]study of tropical forests in that there was no asymptote in the SAR curves (Fig. 1). Though, former studies found the distribution of 90% species (abundance≥20) in Dinhu plot (200000 m^2^) were affected by the terrain factors [76]. However, based on the habitat partitioning theory, there should be an asymptote in the SAR curve, so our results show the habitat partitioning theory is not play leading role in the subtropical forest for small to intermediate scales. We think the lack of an asymptote is the prediction of a spatially explicit, zero-sum, and community drift model that is incorporated with the speciation and the SAR pattern [77], [78]. According to Hubbell’s description of the community, there will always be rare species [75], as, for example, illustrated by the SAD graphs in Fig. 2. As a result, the number of species in the first octaves (number of rare species) are always larger than 1. As in Hubbell’s description [75]], rare species exist in our communities, this also corroborating Hubbell’s theory. But our study can’t approve relative rate of colonization increases while rate of extinction decreases when area increases. So our result can’t prove area *per se* or equilibrium hypothesis.

### Species-abundance Distributions

Preston [41] first formulated the lognormal equation of the SAD by the method of “octaves”, using it to explain the division of resource use by species coexisting in the same community. Whittaker [40] also suggested that certain communities accorded with certain models. The lognormal model is supposed to describe communities where the total number of species is large, and the abundance is determined by many independent factors multiplicatively, whereas the logseries model describes the abundance of species whose diversity is lower or in which the species compete for a single resource [38], [33], [35]. The lognormal model can estimate the theoretical total number of species in the whole community using a truncated distribution [54], while the logseries model cannot be used for this purpose [3]. Using mean *R_a_^2^*-values for comparative performance evaluation, the logcauchy model had the better fit among the three models. Moreover, subtropical forests have high diversity, and their large species number includes many rare species (see Discussion above), which explains why all six communities appeared to be left-side truncated (Fig. 2).

## Conclusions

To conclude, we found that our measures of tree density and diversity varied widely among the three forestry types, with the reasons remaining unclear, which should be subjected of further study. The three SAR models fitted most real data of subtropical forest trees very well for most sampling sizes. But due to intermediate sampling sizes, the logarithm model had the better fit among the three models, especially using randomized sampling. When fitting SADs, the logcauchy model had the better fit among the three tested models. Furthermore, our results suggest that many rare and undiscovered species remain undetected.
